
JOURNAL INFORMATION
==============================
NLM Title Abbreviation: Biospektrum (Heidelb)
Journal ID: Biospektrum (Heidelb)
NLM Journal ID: 9615685

Biospektrum

ISSN: 0947-0867
EISSN: 1868-6249

ARTICLE INFORMATION
==============================
PMCID: PMC8233638
PMID: 34219982
DOI: 10.1007/s12268-021-1590-8
Article ID: 1590
Article version: 1
Subjects: Wissenschaft

Neue Krankheiten mit Bluttranskriptomik entschlüsseln

Ulas Thomas t.ulas@uni-bonn.de
1 2 3
Aschenbrenner Anna C. a.aschenbrenner@uni-bonn.de
1 2 3 4
1 grid.424247.3 0000 0004 0438 0426 Deutsches Zentrum für Neurodegenerative Erkrankungen (DZNE) System-Medizin, Venusberg-Campus 1/99, D-53127 Bonn, Deutschland
2 grid.10388.32 0000 0001 2240 3300 Deutsches Zentrum für Neurodegenerative Erkrankungen (DZNE), Precise Platform for Genomics and Epigenomics at DZNE und Universität Bonn, Bonn, Deutschland
3 grid.10388.32 0000 0001 2240 3300 Genomik & Immunregulation Life & Medical Sciences (LIMES), Institut Rheinische Friedrich-Wilhelms-Universität Bonn, Bonn, Deutschland
4 grid.10417.33 0000 0004 0444 9382 Department of Internal Medicine and Radboud Center for Infectious Diseases (RCI), Radboud University Medical Center, Nijmegen, Niederlande
Electronic publication date: 2021 Jun 26
Print publication date: 2021
Volume: 27
Issue: 4
First page: 372
Last page: 375
Copyright: © Die Autoren 2021
License: Open Access: Dieser Artikel wird unter der Creative Commons Namensnennung 4.0 International Lizenz veröffentlicht, welche die Nutzung, Vervielfältigung, Bearbeitung, Verbreitung und Wiedergabe in jeglichem Medium und Format erlaubt, sofern Sie den/die ursprünglichen Autor(en) und die Quelle ordnungsgemäß nennen, einen Link zur Creative Commons Lizenz beifügen und angeben, ob Änderungen vorgenommen wurden. Die in diesem Artikel enthaltenen Bilder und sonstiges Drittmaterial unterliegen ebenfalls der genannten Creative Commons Lizenz, sofern sich aus der Abbildungslegende nichts anderes ergibt. Sofern das betreffende Material nicht unter der genannten Creative Commons Lizenz steht und die betreffende Handlung nicht nach gesetzlichen Vorschriften erlaubt ist, ist für die oben aufgeführten Weiterverwendungen des Materials die Einwilligung des jeweiligen Rechteinhabers einzuholen. Weitere Details zur Lizenz entnehmen Sie bitte der Lizenzinformation auf http://creativecommons.org/licenses/by/4.0/deed.de.
License URL: https://creativecommons.org/licenses/by/4.0/

issue-copyright-statement: © Springer-Verlag GmbH Deutschland, ein Teil von Springer Nature 2021

==============================
The COVID-19 pandemic is leading to increasing numbers of patients all over the world. Reports on a dysregulated immune system in the severe cases calls for a better characterization of the ongoing changes. To dissect COVID-19-driven immune host responses, we profiled whole blood transcriptomes enabling a data-driven stratification based on molecular phenotype. This analysis allowed prediction of patient subgroup-specific drug candidates targeting the dysregulated systemic immune response of the host.

■ Die RNA-Sequenzierung hat ihren Einzug in klinische Studien gefunden. Transkriptomik von Blutproben ermöglicht die Analyse der zirkulierenden Immunzellen und ist mittlerweile im Kontext von vielen verschiedenen Krankheiten studiert worden. Mit Beginn der COVID-19-Pandemie wurde schnell klar, dass eine SARS-CoV-2-Infektion ganz unterschiedliche klinische Verläufe hervorbringen kann. Während einige COVID-19-Patienten nur über leichte Erkältungssymptome klagen, müssen andere stationär behandelt und beatmet werden. Die schweren Verläufe gehen zudem mit inflammatorischen Parametern einher, die auf eine systemische Immunantwort hinweisen.

Insofern stellten wir uns folgende Fragen:

- Was passiert bei COVID-19?
- Können wir milde und schwere Fälle anhand der Immunreaktion bzw. deren potenzieller Dysregulation im Blut auslesen?
- Lassen sich COVID-19-Patienten anhand ihrer Bluttranskriptome stratifizieren?
- Können die Transkriptomdaten genutzt werden, um Vorhersagen für potenzielle neue Therapieansätze zu treffen?

## Neutrophilen-assoziierte Signaturen bestimmen das COVID-19-Bluttranskriptom

Über unsere klinischen Partner in Athen, Nijmegen und Bonn hatten wir Zugang zu Blutproben von drei verschiedenen Kohorten von COVID-19-Patienten unterschiedlichen Schweregrades, welche wir mittels RNA-Sequenzierung untersuchten [1]. Die RNA-

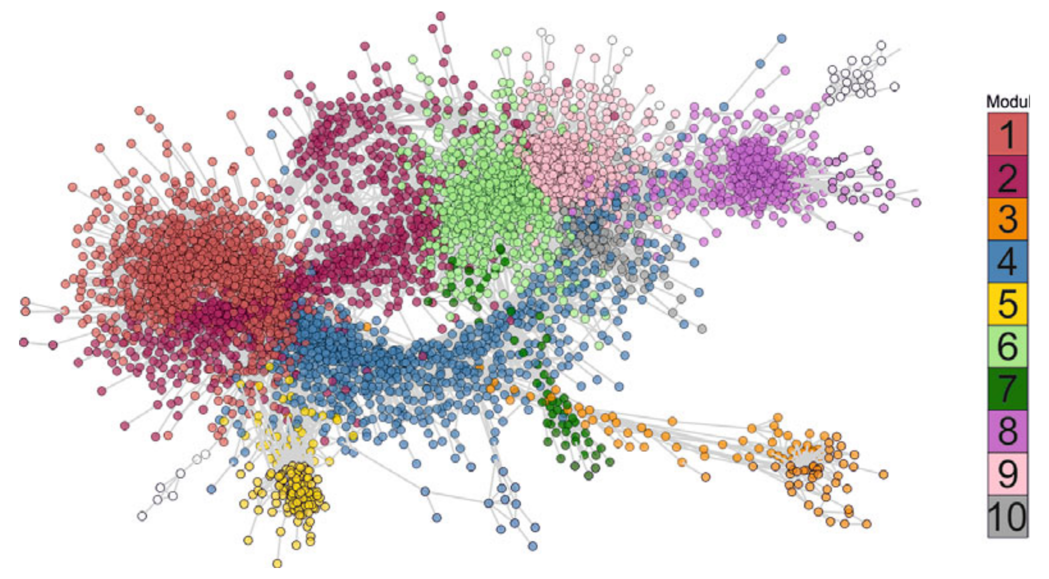

◀ **Abb. 1:** Koexpressionsnetzwerk aus 6.085 Genen. Die 10 identifizierten Module werden durch unterschiedliche Farben dargestellt. Gene werden als Knoten und eine Korrelation zwischen zwei Genen durch eine Verbindung dargestellt.

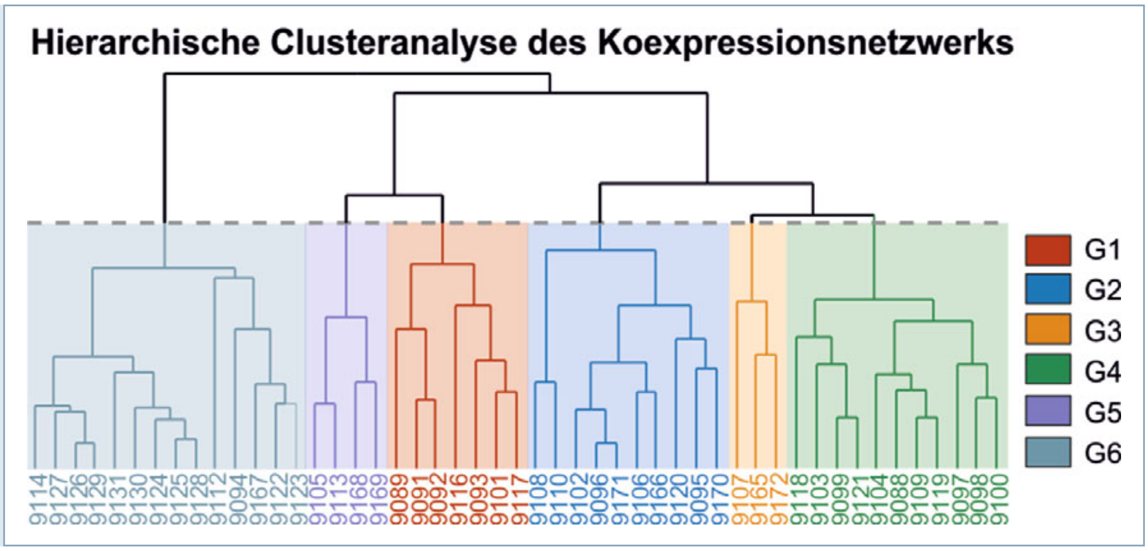

▶ **Abb. 2:** Hierarchische Gruppierung der Proben mit den 6 Patientengruppen G1–G6.

Sequenzierung erfasst dabei in etwa 15.000 Gene und bestimmt gleichzeitig deren Quantität. Im ersten Teil unserer Studie haben wir zunächst die sequenzierten Transkriptome von 39 COVID-19-Patienten und zehn Kontrollspendern mithilfe eines klassischen bioinformatischen Ansatzes untersucht.

Im klassischen Ansatz werden die Daten mit den klinischen Daten – wie Krankheitsschweregrad, WHO-Klassifizierung, Alter, Geschlecht usw. – verknüpft und wissensorientiert analysiert. Dabei werden die Transkriptomdaten mithilfe der klinischen Daten gruppiert und Unterschiede zwischen diesen Gruppen bestimmt. Der Vergleich zwischen den COVID-19-Patienten und den Kontrollspendern ergab 2.289 hoch- und 912 herunterregulierte differenziell exprimierte Gene. Im darauffolgenden Schritt haben wir die Gruppe der COVID-19-Patienten in solche mit mildem und schwerem Verlauf unterteilt. Viele der im Vergleich zwischen COVID-19 und den Kontrollspendern gefundenen differenziell exprimierten Gene wurden auch bei der zusätzlichen Unterscheidung nach Schweregrad gefunden. Sowohl Patienten mit schwerem als auch mit mildem COVID-19-Verlauf zeigten eine erhöhte Expression der Neutrophilen-spezifischen Gene *CD177* und *HP* im Vergleich zu Kontrollspendern. Unter den am stärksten differenziell niedriger exprimierten Genen waren Lymphozyten-assoziierte Gene wie *NELL2*, *RCAN3*, *RORC*, *BACH2* und *KLRB1*. Auch funktionelle Vorhersagen der differenziell exprimierten Gene zeigte sehr deutlich, dass Neutrophile eine prominente Funktion während COVID-19 spielen. Der Vergleich von milden und schweren Verläufen zeigte zudem stärkere Veränderungen mit zunehmendem Schweregrad der Erkrankung.

## Koexpressionsnetzwerkanalyse offenbart Subtypen mit distinkten molekularen Signaturen

Der an den klinischen Daten orientierte klassische Ansatz der Analyse half allerdings nicht dabei, alle in den Daten vorhandenen Strukturen zu erklären. Daher entschieden wir uns, die Transkriptome der Patienten und Kontrollspender zusätzlich mithilfe eines Koexpressionsnetzwerks zu analysieren (https://github.com/Ulas-lab/CoCena2). Die Koexpressionsnetzwerkanalyse ermöglicht die gleichzeitige Identifizierung, Gruppierung und Erforschung von Tausenden von Genen mit ähnlichen Expressionsmustern. Dabei wird keinerlei *a priori* Wissen benötigt, um das Netzwerk zu generieren und Substrukturen (Module) im Netzwerk zu identifizieren. Diese Analyse ergab ein Netzwerk aus 6.085 Genen und zehn Modulen (**Abb. 1**). Als nächstes haben wir mithilfe des Netzwerks die Ähnlichkeit der einzelnen Patienten und Kontrollen untereinander untersucht. Dabei ergab die Ähnlichkeitsanalyse ein Dendrogramm, das auf mehr als nur drei Gruppen (milder und schwerer Verlauf und Kontrollen) hindeutete. Die Berechnung des Silhouettenkoeffizients bestätigte diese Vermutung und schlug sechs Gruppen als optimale Anzahl in unserem Datensatz vor (**Abb. 2**).

Die so identifizierten fünf unterschiedlichen COVID-19-assoziierten Patientengruppen (G1–G5) sind nur teilweise durch den Schweregrad der Erkrankung zu erklären und veranschaulichen sehr deutlich die Heterogenität der Immunantwort der COVID-19-Patienten. Die Vorhersage der genassoziierten Funktionen der jeweiligen Gene in den zehn Modulen sollte dabei helfen, die fünf COVID-19-Patientengruppen und Kontrollspender besser zu verstehen. Zum Beispiel wurden entzündungsassoziierte Gensignaturen für die Patientengruppen G1 und G2 in unterschiedlichem Ausmaß vorhergesagt, was darauf hinweist, dass in diesen Patientengruppen möglicherweise eine stärkere entzündliche Immunreaktion vonstattengeht (**Abb. 3**). Nur ein geringer Anstieg des entzündungsassoziierten Moduls 2 und ein Anstieg der Expression in den Genen von Modul 3 (angereichert mit oxidativer Phosphorylierung, mTORC1-Signaling und zellzyklusassoziierten Gensignaturen) beschreiben die Patientengruppe G4. Die Kontrollpatientengruppe G6 war nicht mit entzündlichen Prozessen assoziiert und zeigte eine Anreicherung von grundlegenden zellulären und metabolischen Prozessen. Besonders die Analyse des Moduls 6 (spezifisch für G1/G2) zeigte eine deutliche Anreicherung von Gensignaturen, die im Zusammenhang mit der Aktivierung von Granulozyten/Neutrophilen stehen. Die Analyse der im Blut zirkulierenden Immunzellpopulationen mittels Durchflusszytometrie zeigte erhöhte Neutrophilen/Lymphozyten-Anteile insbesondere für G1 und G2, was zuvor bereits mit schweren Verläufen in Verbindung gebracht worden war [2].

## Granulozyten zeigen transkriptionelle Veränderungen bei schwerem COVID-19-Verlauf

Um der Ursache der im Vollblut von Patienten – insbesondere mit schwerem COVID-19-Verlauf – beobachteten Neutrophilen-

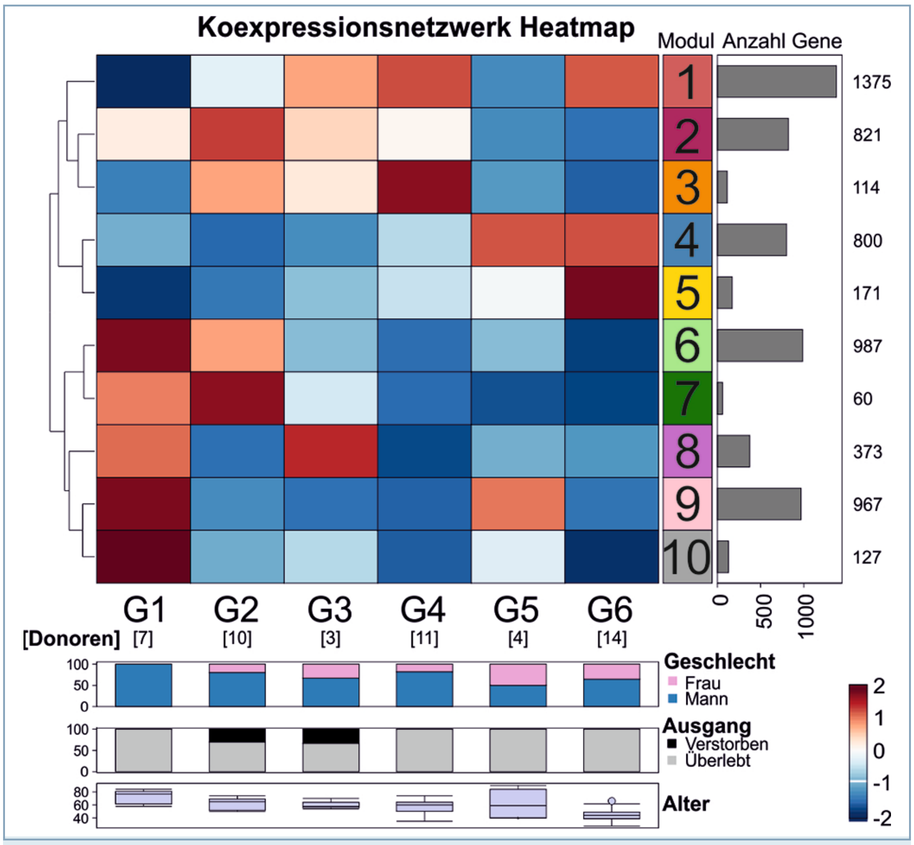

▲ **Abb. 3:** Heatmap des Koexpressionsnetzwerkes mit den 10 identifizierten Modulen und den 6 datengetriebenen Patientengruppen.

assoziierten Aktivierungssignaturen auf den Grund zu gehen, sequenzierten wir Transkriptome von Granulozyten einer zweiten longitudinal angelegten Kohorte. Diese Analyse zeigte, dass es neben der relativen Zunahme von Neutrophilen in schweren COVID-19-Fällen tatsächlich Veränderungen im Transkriptionsprogramm dieser Zellen selbst gibt. Wir fanden eine Anreicherung von Signaturen, die typisch für Vorläuferzellen von Neutrophilen sind [3], und Hinweise auf gleichzeitige entzündliche und suppressive Eigenschaften, was für eine Dysregulation im peripheren Granulozytenkompartiment spricht. Interessanterweise sind diese Granulozyten-Phänotpyen auch in der Analyse der Vollbluttranskriptome offensichtlich.

## COVID-19 im Kontext anderer Krankheiten

Um COVID-19 in den Kontext anderer bekannter Krankheiten zu stellen, fassten wir als nächstes Transkriptome von zwölf weiteren Krankheiten, darunter verschiedene virale und bakterielle Infektionen sowie immunologische Erkrankungen, in einem großen Datensatz zusammen. Dieser umfasste 3.176 Patientenproben inklusive unserer 39 COVID-19-Patientenproben, wurde auf unser COVID-19-Koexpressionsnetzwerk projiziert und die zehn bekannten Module über die verschiedenen Krankheiten hinweg verglichen. Gene des Moduls 6 waren in unserer Patientengruppe G1 (schwerer COVID-19-Verlauf) sowie bei Sepsis, bei Patienten mit Influenza A und mit Tuberkulose und HIV-Infektion am stärksten exprimiert, weniger jedoch bei individuell auftretender HIV-Infektion oder Tuberkulose. Von vielen der Gene innerhalb des Moduls 6 ist bekannt, dass sie mit der Induktion von NETs (*neutrophil extracellular traps*) in Zusammenhang stehen (z. B. *PKC*, *PADI4*, *LTB4*) und diese wiederum stehen auch im Zusammenhang mit schweren Verläufen bei COVID-19 und Sepsis (Blutvergiftung). Obwohl wir diese und andere Überschneidungen von Modulen zwischen COVID-19 und mehreren anderen infektiösen und immunologischen Erkrankungen beobachten konnten, war jede unserer molekular definierten COVID-19-Patientengruppen (G1–G5) durch eine spezifische Kombination dieser Module charakterisiert. Dies weist eindeutig auf eine einzigartige, durch die SARS-CoV-2-Infektion vermittelte. Immunantwort hin, die bei der Entwicklung von patientenorientierten Therapien berücksichtigt werden muss.

## Patientengruppen-orientierte Vorhersage von potenziellen Therapiestrategien

Trotz der immunologisch getriebenen Natur von schwerem COVID-19 fokussierten sich die ersten klinischen Studien auf Medikamente gegen das Virus selbst und seine direkten Interaktionspartner. Bei der Untersuchung von für andere Indikationen zugelassenen und bei COVID-19 in der klinischen Prüfung befindlichen Medikamenten (zur Zeit unserer Studie im Frühjahr 2020) und deren Zielgenen, konnten wir nur 162 dieser Zielgene in unserem Koexpressionsnetzwerk wiederfinden. Das bedeutete, dass viele der spezifisch regulierten Gene in unseren Patientengruppen von keinem aus COVID-19-Studien bekannten Medikamenten beeinflusst werden konnten. Aus diesem Grund entwickelten wir einen Ansatz, um für jede unserer fünf Patientengruppen bekannte Medikamente zu identifizieren, die theoretisch in der Lage sein sollten, die jeweiligen spezifischen Gensignaturen derjenigen anzunähern, die wir bei Kontrollspendern gefunden hatten. Unter Verwendung von Patientengruppen-spezifischen differenziell exprimierten Genen in Kombination mit den Medikamentendatenbanken *NIH Library of Integrated Network-Based Cellular Signatures* (iLINCS) und des *Repurposing Hub* des Broad Instituts haben wir nach Substanzen gesucht, die eine umgekehrte Signatur in menschlichen Zellen hervorrufen. Diese Suche ergab 63.000 Signaturen von 940 bekannten Verbindungen/Wirkstoffen, die von uns weiterverarbeitet wurden. Mithilfe von Signaturanreicherungsanalysen konnten wir modellieren, wie stark diese 63.000 Signaturen unsere Patientengruppen-spezifischen Signaturen umkehren können und konnten diese dann nach potenzieller Wirksamkeit gruppieren. So konnten wir bereits im März 2020 Dexamethason als potenziell wirksames Therapeutikum für die G1-Patientengruppe vorhersagen. Wenige Monate später berichteten die Autoren der RECOVERY-Studie über eine um 28 Tage reduzierte Mortalität in einer Subgruppe von Patienten mit schwerem COVID-19, die beatmet werden müssen – nicht aber in solchen, die keine Sauerstoffgabe nötig hatten [4].

Unser Ansatz zeigt, wie molekulare Signaturen von Bluttranskriptom-basierten stratifizierten Patientengruppen in einem *in silico*-Verfahren genutzt werden können, um potenziell wirksame Medikamente gegen eine noch unbekannte Krankheit zu identifizieren. Viele der von uns identifizierten Treffer werden inzwischen in klinischen Studien getestet.

## Ausblick

COVID-19 beginnt als lokale respiratorische Infektion mit SARS-CoV-2, die im weiteren Verlauf eine systemische Immunantwort induziert, die v. a. in schweren Krankheitsverläufen eine Dysregulation der Immunantwort aufweist, die auch als virale Sepsis beschrieben wurde [5].

Unsere hier vorgestellte Studie zeigt, dass sich Transkriptome aus dem Blut hervorragend dazu eignen, die Pathophysiologie von COVID-19 besser zu verstehen. Die daraus resultierende Patientenstratifizierung ermöglicht eine genauere Analyse ihrer Immunphänotypen. Des Weiteren wurde deutlich, dass eine derartige Einteilung von Patienten in klinischen Studien genutzt werden könnte, um Behandlungsstrategien im Sinne der Präzisionsmedizin gezielter in Abhängigkeit von den molekularen Signaturen der Patienten zu evaluieren.


Funding note: Open Access funding enabled and organized by Projekt DEAL.

Danksagung

Wir danken unseren klinischen Partnern Prof. Dr. Evangelos Giamarellos-Bourboulis (Attikon Universitätsklinikum, Athen, Griechenland), Prof. Dr. Mihai Netea und Dr. Matthijs Kox (Radboud umc, Nijmegen, Niederlande) sowie Prof. Dr. Jacob Nattermann (Uniklinikum Bonn). Des Weiteren möchten wir unseren Ko-Autoren für ihren Beitrag danken, insbesondere unserem gesamten Team, das sich unter besonderen Umständen mit Enthusiasmus dieser Studie gewidmet hat und Prof. Dr. Joachim Schultze für seinen Einsatz und Unterstützung, v. a. durch die Initiation und Belebung der Deutschen COVID-19 OMICS Initiative (DeCOI).

Thomas Ulas 2003–2009 Studium der Bioinformatik und Genomforschung (B.Sc.) und der Genombasierten Systembiologie (M.Sc.). 2009–2012 Promotion in der Bioinformatik. 2012–2015 Postdoktorand an der Universität Bonn. 2015–2018 Team Leader Bioinformatics im Labor von Prof. Dr. J. L. Schultze. Seit 2018 Team Leader PRECISE bioinformatics, System-Medizin, Deutsches Zentrum für Neurodegenerative Erkrankungen.

Anna Aschenbrenner 2003–2014 Studium der Molekularen Biomedizin an der Universität Bonn mit anschließender Promotion. 2014–2016 Postdoktorandin an der Universität Bonn im Labor von Prof. Dr. Dr. h.c. M. Hoch. 2016–2019 Postdoktorandin an der Universität Bonn im Labor von Prof. Dr. J. L. Schultze. Seit 2019 Team Leader Clinical Single Cell Omics, System-Medizin, Deutsches Zentrum für Neurodegenerative Erkrankungen.


Literatur

[1] Aschenbrenner AC Mouktaroudi M Krämer B Disease severity-specific neutrophil signatures in blood transcriptomes stratify COVID-19 patients Genome Med 2021 13 7 10.1186/s13073-020-00823-5 33441124 PMC7805430
[2] Reusch N De Domenico E Bonaguro L Neutrophils in COVID-19 Front Immunol 2021 12 652470 10.3389/fimmu.2021.652470 33841435 PMC8027077
[3] Schulte-Schrepping J Reusch N Paclik D Severe COVID-19 is marked by a dysregulated myeloid cell compartment Cell 2020 182 1419 1440 10.1016/j.cell.2020.08.001 32810438 PMC7405822
[4] RECOVERY Collaborative GroupHorby P Lim WS Dexamethasone in hospitalized patients with Covid-19 N Engl J Med 2021 384 693 704 10.1056/NEJMoa2021436 32678530 PMC7383595
[5] Schultze JL Aschenbrenner AC COVID-19 and the human innate immune system Cell 2021 184 1671 1692 10.1016/j.cell.2021.02.029 33743212 PMC7885626
